# 2-[(3,5-Di-*tert*-butyl-4-hydroxy­benz­yl)­sulfan­yl]-*N*′-isopropyl­ideneaceto­hydrazide

**DOI:** 10.1107/S1600536809007843

**Published:** 2009-03-11

**Authors:** Wagee A. Yehye, Azhar Ariffin, Seik Weng Ng

**Affiliations:** a Department of Chemistry, University of Malaya, 50603 Kuala Lumpur, Malaysia

## Abstract

The title compound, C_20_H_32_N_2_O_2_S, the condensation product of a thio­acetohydrazine and acetone, has a two-coordinate S atom and the angle at this atom is 100.7 (1)°. The (CH_3_)C=N—NH—C(O)– substituent engages in N—H⋯O hydrogen-bonding inter­actions with the substituent of an adjacent mol­ecule across a center of inversion, generating a dimeric structure.

## Related literature

There are several structural studies of (CH_3_)C=N–NH–C(O)–*X* compounds; for *N*-acetyl-*N*′-isopropyl­idenehydrazine, see: Khusainova *et al.* (2004 ▶). For the synthesis of the thio­acetohydrazine reactant, see: MacLeay & Meyers (1989 ▶); Myers & MacLeay (1989 ▶).
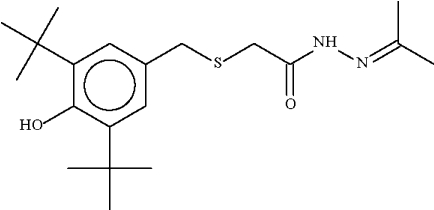


## Experimental

### 

#### Crystal data

C_20_H_32_N_2_O_2_S
*M* *_r_* = 364.54
Monoclinic, 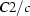

*a* = 30.8643 (10) Å
*b* = 10.0128 (3) Å
*c* = 13.9596 (5) Å
β = 96.227 (2)°
*V* = 4288.6 (2) Å^3^
*Z* = 8
Mo *K*α radiation
μ = 0.17 mm^−1^
*T* = 100 K
0.25 × 0.15 × 0.10 mm

#### Data collection

Bruker SMART APEX diffractometer
Absorption correction: multi-scan (*SADABS*; Sheldrick, 1996 ▶) *T*_min_ = 0.960, *T*_max_ = 0.984
14652 measured reflections
4886 independent reflections
3240 reflections with *I* > 2σ(*I*)
*R*_int_ = 0.052

#### Refinement

*R*[*F* ^2^ > 2σ(*F* ^2^)] = 0.047
*wR*(*F* ^2^) = 0.135
*S* = 1.05
4886 reflections
237 parameters
H-atom parameters constrained
Δρ_max_ = 0.31 e Å^−3^
Δρ_min_ = −0.28 e Å^−3^

### 

Data collection: *APEX2* (Bruker, 2007 ▶); cell refinement: *SAINT* (Bruker, 2007 ▶); data reduction: *SAINT*; program(s) used to solve structure: *SHELXS97* (Sheldrick, 2008 ▶); program(s) used to refine structure: *SHELXL97* (Sheldrick, 2008 ▶); molecular graphics: *X-SEED* (Barbour, 2001 ▶); software used to prepare material for publication: *publCIF* (Westrip, 2009 ▶).

## Supplementary Material

Crystal structure: contains datablocks global, I. DOI: 10.1107/S1600536809007843/tk2379sup1.cif

Structure factors: contains datablocks I. DOI: 10.1107/S1600536809007843/tk2379Isup2.hkl

Additional supplementary materials:  crystallographic information; 3D view; checkCIF report

## Figures and Tables

**Table 1:** Hydrogen-bond geometry (Å, °)

*D*—H⋯*A*	*D*—H	H⋯*A*	*D*⋯*A*	*D*—H⋯*A*
N1—H1⋯O2^i^	0.88	2.10	2.940 (2)	159

Symmetry code: (i) 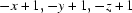.

**Fig. 1.**
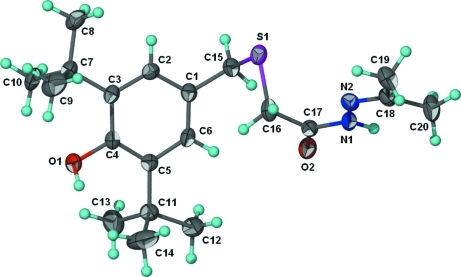
Thermal ellipsoid plot (Barbour, 2001) of C20H32N2O2S at the 70% probability level. Hydrogen atoms are drawn as spheres of arbitrary radii.

## supplementary crystallographic information

### Experimental

2-(3,5-Di-*tert*-butyl-4-hydroxybenzylthio)acetohydrazine (0.5 g, 1.54 mmol) and acetone (10 ml) were heated for 6 h; several drops of acetic acid were added to the reaction. The solvent was removed and the product recrystallized from hexane.

### Refinement

Carbon-bound H-atoms were placed in calculated positions (C–H 0.95–0.99 Å) and were included in the refinement in the riding model approximation with *U*(H) set to 1.2–1.5*U*(C). The oxygen- and nitrogen-bound H-atoms were similarly treated (O–H 0.84 and N–H 0.88 Å).

The hydroxy H-atom does not form a hydrogen bond; it is probably disordered over several positions. In one position, it is less than 2 Å from a hydrogen atom of the C14 methyl group. The two *tert*-butyl groups are probably also disordered, but the disorder could not be resolved into multiple positions.

### Crystal data

**Table:**

C_20_H_32_N_2_O_2_S	*F*(000) = 1584
*M**_r_* = 364.54	*D*_x_ = 1.129 Mg m^−^^3^
Monoclinic, *C*2/*c*	Mo *K*α radiation, λ = 0.71073 Å
Hall symbol: -C 2yc	Cell parameters from 1963 reflections
*a* = 30.8643 (10) Å	θ = 2.6–26.3°
*b* = 10.0128 (3) Å	µ = 0.17 mm^−^^1^
*c* = 13.9596 (5) Å	*T* = 100 K
β = 96.227 (2)°	Block, colorless
*V* = 4288.6 (2) Å^3^	0.25 × 0.15 × 0.10 mm
*Z* = 8	

### Data collection

**Table:**

Bruker SMART APEX diffractometer	4886 independent reflections
Radiation source: fine-focus sealed tube	3240 reflections with *I* > 2σ(*I*)
graphite	*R*_int_ = 0.052
ω scans	θ_max_ = 27.5°, θ_min_ = 1.3°
Absorption correction: multi-scan (*SADABS*; Sheldrick, 1996)	*h* = −39→40
*T*_min_ = 0.960, *T*_max_ = 0.984	*k* = −13→13
14652 measured reflections	*l* = −18→14

### Refinement

**Table:**

Refinement on *F*^2^	Primary atom site location: structure-invariant direct methods
Least-squares matrix: full	Secondary atom site location: difference Fourier map
*R*[*F*^2^ > 2σ(*F*^2^)] = 0.047	Hydrogen site location: inferred from neighbouring sites
*wR*(*F*^2^) = 0.135	H-atom parameters constrained
*S* = 1.05	*w* = 1/[σ^2^(*F*_o_^2^) + (0.0627*P*)^2^ + 0.8959*P*] where *P* = (*F*_o_^2^ + 2*F*_c_^2^)/3
4886 reflections	(Δ/σ)_max_ = 0.001
237 parameters	Δρ_max_ = 0.31 e Å^−^^3^
0 restraints	Δρ_min_ = −0.28 e Å^−^^3^

### Fractional atomic coordinates and isotropic or equivalent isotropic displacement parameters (Å^2^)

**Table:**

	*x*	*y*	*z*	*U*_iso_*/*U*_eq_	
S1	0.394869 (18)	0.18708 (5)	0.52819 (4)	0.02810 (16)	
O1	0.32415 (5)	0.04431 (14)	0.08151 (10)	0.0287 (4)	
H1O	0.3479	0.0304	0.0591	0.066 (10)*	
O2	0.45752 (5)	0.41852 (14)	0.43360 (10)	0.0284 (4)	
N1	0.49822 (5)	0.32105 (16)	0.55791 (12)	0.0228 (4)	
H1	0.5109	0.3970	0.5762	0.033 (6)*	
N2	0.51037 (5)	0.20268 (16)	0.60634 (12)	0.0233 (4)	
C1	0.34695 (6)	0.22530 (19)	0.35011 (14)	0.0212 (4)	
C2	0.31851 (6)	0.11801 (18)	0.33503 (15)	0.0208 (4)	
H2	0.3045	0.0851	0.3876	0.025*	
C3	0.30992 (6)	0.05737 (18)	0.24556 (15)	0.0205 (4)	
C4	0.33196 (6)	0.10754 (18)	0.17019 (14)	0.0207 (4)	
C5	0.35919 (6)	0.21967 (19)	0.18074 (14)	0.0206 (4)	
C6	0.36638 (6)	0.27540 (19)	0.27299 (14)	0.0213 (4)	
H6	0.3853	0.3502	0.2830	0.026*	
C7	0.27708 (7)	−0.0579 (2)	0.22932 (15)	0.0257 (5)	
C8	0.25474 (7)	−0.0877 (2)	0.31963 (16)	0.0326 (5)	
H8A	0.2394	−0.0077	0.3383	0.049*	
H8B	0.2767	−0.1138	0.3723	0.049*	
H8C	0.2338	−0.1606	0.3061	0.049*	
C9	0.30019 (9)	−0.1865 (2)	0.2033 (2)	0.0425 (6)	
H9A	0.2790	−0.2594	0.1946	0.064*	
H9B	0.3229	−0.2093	0.2554	0.064*	
H9C	0.3135	−0.1729	0.1435	0.064*	
C10	0.24093 (7)	−0.0210 (2)	0.14912 (17)	0.0360 (6)	
H10A	0.2260	0.0600	0.1674	0.054*	
H10B	0.2199	−0.0945	0.1398	0.054*	
H10C	0.2538	−0.0049	0.0890	0.054*	
C11	0.38057 (7)	0.2813 (2)	0.09618 (16)	0.0284 (5)	
C12	0.40558 (9)	0.4089 (2)	0.12623 (17)	0.0404 (6)	
H12A	0.4293	0.3879	0.1764	0.061*	
H12B	0.3858	0.4734	0.1513	0.061*	
H12C	0.4177	0.4472	0.0703	0.061*	
C13	0.34528 (10)	0.3206 (3)	0.01478 (18)	0.0480 (7)	
H13A	0.3255	0.3861	0.0390	0.072*	
H13B	0.3287	0.2410	−0.0078	0.072*	
H13C	0.3591	0.3596	−0.0387	0.072*	
C14	0.41348 (10)	0.1852 (2)	0.0594 (2)	0.0544 (8)	
H14A	0.4342	0.1558	0.1135	0.082*	
H14B	0.4292	0.2307	0.0116	0.082*	
H14C	0.3981	0.1075	0.0297	0.082*	
C15	0.35698 (7)	0.2863 (2)	0.44856 (14)	0.0242 (5)	
H15A	0.3295	0.2968	0.4783	0.029*	
H15B	0.3695	0.3764	0.4417	0.029*	
C16	0.44235 (7)	0.18946 (19)	0.46239 (16)	0.0258 (5)	
H16A	0.4615	0.1128	0.4823	0.031*	
H16B	0.4332	0.1816	0.3924	0.031*	
C17	0.46677 (6)	0.3174 (2)	0.48258 (14)	0.0221 (4)	
C18	0.54119 (7)	0.2084 (2)	0.67522 (15)	0.0252 (5)	
C19	0.55491 (8)	0.0790 (2)	0.72261 (16)	0.0328 (5)	
H19A	0.5361	0.0070	0.6946	0.049*	
H19B	0.5524	0.0852	0.7919	0.049*	
H19C	0.5852	0.0600	0.7124	0.049*	
C20	0.56467 (8)	0.3315 (2)	0.71190 (17)	0.0376 (6)	
H20A	0.5435	0.4023	0.7203	0.056*	
H20B	0.5844	0.3607	0.6655	0.056*	
H20C	0.5816	0.3124	0.7739	0.056*	

### Atomic displacement parameters (Å^2^)

**Table:**

	*U*^11^	*U*^22^	*U*^33^	*U*^12^	*U*^13^	*U*^23^
S1	0.0292 (3)	0.0299 (3)	0.0231 (3)	−0.0090 (2)	−0.0063 (2)	0.0075 (2)
O1	0.0321 (9)	0.0280 (8)	0.0264 (9)	−0.0086 (7)	0.0057 (7)	−0.0100 (6)
O2	0.0270 (8)	0.0279 (8)	0.0286 (9)	−0.0029 (6)	−0.0044 (7)	0.0037 (6)
N1	0.0219 (9)	0.0237 (8)	0.0219 (9)	−0.0034 (7)	−0.0018 (7)	−0.0016 (7)
N2	0.0237 (9)	0.0257 (9)	0.0203 (9)	0.0017 (7)	0.0019 (7)	0.0006 (7)
C1	0.0188 (10)	0.0232 (9)	0.0207 (11)	0.0016 (8)	−0.0024 (8)	0.0014 (8)
C2	0.0174 (10)	0.0224 (9)	0.0223 (11)	0.0006 (8)	0.0009 (8)	0.0044 (8)
C3	0.0170 (10)	0.0193 (9)	0.0243 (11)	0.0003 (8)	−0.0015 (8)	0.0014 (8)
C4	0.0200 (10)	0.0199 (9)	0.0214 (11)	−0.0004 (8)	−0.0012 (8)	−0.0024 (8)
C5	0.0208 (10)	0.0201 (9)	0.0208 (11)	0.0006 (8)	0.0015 (8)	0.0007 (8)
C6	0.0190 (10)	0.0193 (9)	0.0244 (11)	−0.0026 (8)	−0.0031 (8)	0.0013 (8)
C7	0.0237 (11)	0.0250 (10)	0.0282 (12)	−0.0071 (9)	0.0023 (9)	−0.0018 (9)
C8	0.0303 (12)	0.0305 (11)	0.0373 (14)	−0.0113 (10)	0.0048 (10)	0.0031 (10)
C9	0.0496 (16)	0.0223 (11)	0.0580 (18)	−0.0087 (11)	0.0172 (13)	−0.0045 (11)
C10	0.0269 (12)	0.0433 (13)	0.0359 (14)	−0.0155 (11)	−0.0046 (10)	−0.0016 (11)
C11	0.0367 (13)	0.0260 (11)	0.0232 (12)	−0.0095 (9)	0.0066 (10)	0.0000 (9)
C12	0.0553 (16)	0.0360 (13)	0.0310 (14)	−0.0208 (12)	0.0096 (12)	0.0026 (10)
C13	0.0673 (19)	0.0478 (15)	0.0261 (14)	−0.0202 (14)	−0.0072 (13)	0.0108 (11)
C14	0.0605 (18)	0.0398 (14)	0.071 (2)	−0.0102 (13)	0.0424 (16)	−0.0037 (14)
C15	0.0231 (11)	0.0274 (10)	0.0214 (11)	−0.0001 (9)	−0.0013 (9)	0.0000 (8)
C16	0.0252 (11)	0.0233 (10)	0.0270 (12)	0.0028 (9)	−0.0065 (9)	−0.0036 (9)
C17	0.0181 (10)	0.0275 (10)	0.0207 (11)	−0.0004 (8)	0.0024 (8)	−0.0024 (9)
C18	0.0276 (11)	0.0279 (11)	0.0199 (11)	0.0001 (9)	0.0018 (9)	−0.0020 (8)
C19	0.0395 (13)	0.0323 (12)	0.0253 (13)	0.0048 (10)	−0.0031 (10)	−0.0029 (9)
C20	0.0441 (14)	0.0353 (12)	0.0294 (13)	−0.0072 (11)	−0.0145 (11)	0.0031 (10)

### Geometric parameters (Å, °)

**Table:**

S1—C16	1.812 (2)	C10—H10A	0.9800
S1—C15	1.819 (2)	C10—H10B	0.9800
O1—C4	1.388 (2)	C10—H10C	0.9800
O1—H1O	0.8400	C11—C14	1.528 (3)
O2—C17	1.237 (2)	C11—C12	1.527 (3)
N1—C17	1.352 (2)	C11—C13	1.537 (3)
N1—N2	1.396 (2)	C12—H12A	0.9800
N1—H1	0.8800	C12—H12B	0.9800
N2—C18	1.278 (3)	C12—H12C	0.9800
C1—C6	1.382 (3)	C13—H13A	0.9800
C1—C2	1.389 (3)	C13—H13B	0.9800
C1—C15	1.505 (3)	C13—H13C	0.9800
C2—C3	1.388 (3)	C14—H14A	0.9800
C2—H2	0.9500	C14—H14B	0.9800
C3—C4	1.406 (3)	C14—H14C	0.9800
C3—C7	1.536 (3)	C15—H15A	0.9900
C4—C5	1.401 (3)	C15—H15B	0.9900
C5—C6	1.399 (3)	C16—C17	1.498 (3)
C5—C11	1.542 (3)	C16—H16A	0.9900
C6—H6	0.9500	C16—H16B	0.9900
C7—C8	1.531 (3)	C18—C20	1.493 (3)
C7—C9	1.535 (3)	C18—C19	1.495 (3)
C7—C10	1.537 (3)	C19—H19A	0.9800
C8—H8A	0.9800	C19—H19B	0.9800
C8—H8B	0.9800	C19—H19C	0.9800
C8—H8C	0.9800	C20—H20A	0.9800
C9—H9A	0.9800	C20—H20B	0.9800
C9—H9B	0.9800	C20—H20C	0.9800
C9—H9C	0.9800		
			
C16—S1—C15	100.65 (10)	C14—C11—C5	111.09 (18)
C4—O1—H1O	109.5	C12—C11—C5	111.70 (17)
C17—N1—N2	119.11 (16)	C13—C11—C5	109.91 (19)
C17—N1—H1	120.4	C11—C12—H12A	109.5
N2—N1—H1	120.4	C11—C12—H12B	109.5
C18—N2—N1	117.74 (17)	H12A—C12—H12B	109.5
C6—C1—C2	118.89 (18)	C11—C12—H12C	109.5
C6—C1—C15	120.02 (18)	H12A—C12—H12C	109.5
C2—C1—C15	121.09 (18)	H12B—C12—H12C	109.5
C3—C2—C1	122.04 (19)	C11—C13—H13A	109.5
C3—C2—H2	119.0	C11—C13—H13B	109.5
C1—C2—H2	119.0	H13A—C13—H13B	109.5
C2—C3—C4	117.15 (17)	C11—C13—H13C	109.5
C2—C3—C7	121.32 (18)	H13A—C13—H13C	109.5
C4—C3—C7	121.52 (17)	H13B—C13—H13C	109.5
O1—C4—C5	120.24 (17)	C11—C14—H14A	109.5
O1—C4—C3	116.94 (17)	C11—C14—H14B	109.5
C5—C4—C3	122.76 (18)	H14A—C14—H14B	109.5
C6—C5—C4	116.74 (18)	C11—C14—H14C	109.5
C6—C5—C11	120.44 (17)	H14A—C14—H14C	109.5
C4—C5—C11	122.82 (17)	H14B—C14—H14C	109.5
C1—C6—C5	122.24 (18)	C1—C15—S1	113.01 (14)
C1—C6—H6	118.9	C1—C15—H15A	109.0
C5—C6—H6	118.9	S1—C15—H15A	109.0
C8—C7—C9	107.06 (18)	C1—C15—H15B	109.0
C8—C7—C3	111.95 (17)	S1—C15—H15B	109.0
C9—C7—C3	110.44 (17)	H15A—C15—H15B	107.8
C8—C7—C10	106.88 (18)	C17—C16—S1	109.51 (14)
C9—C7—C10	110.41 (19)	C17—C16—H16A	109.8
C3—C7—C10	110.01 (17)	S1—C16—H16A	109.8
C7—C8—H8A	109.5	C17—C16—H16B	109.8
C7—C8—H8B	109.5	S1—C16—H16B	109.8
H8A—C8—H8B	109.5	H16A—C16—H16B	108.2
C7—C8—H8C	109.5	O2—C17—N1	120.74 (18)
H8A—C8—H8C	109.5	O2—C17—C16	120.97 (18)
H8B—C8—H8C	109.5	N1—C17—C16	118.26 (18)
C7—C9—H9A	109.5	N2—C18—C20	126.07 (19)
C7—C9—H9B	109.5	N2—C18—C19	116.51 (19)
H9A—C9—H9B	109.5	C20—C18—C19	117.41 (19)
C7—C9—H9C	109.5	C18—C19—H19A	109.5
H9A—C9—H9C	109.5	C18—C19—H19B	109.5
H9B—C9—H9C	109.5	H19A—C19—H19B	109.5
C7—C10—H10A	109.5	C18—C19—H19C	109.5
C7—C10—H10B	109.5	H19A—C19—H19C	109.5
H10A—C10—H10B	109.5	H19B—C19—H19C	109.5
C7—C10—H10C	109.5	C18—C20—H20A	109.5
H10A—C10—H10C	109.5	C18—C20—H20B	109.5
H10B—C10—H10C	109.5	H20A—C20—H20B	109.5
C14—C11—C12	106.5 (2)	C18—C20—H20C	109.5
C14—C11—C13	110.9 (2)	H20A—C20—H20C	109.5
C12—C11—C13	106.61 (18)	H20B—C20—H20C	109.5
			
C17—N1—N2—C18	177.73 (18)	C4—C3—C7—C9	65.2 (3)
C6—C1—C2—C3	1.8 (3)	C2—C3—C7—C10	122.0 (2)
C15—C1—C2—C3	−177.81 (18)	C4—C3—C7—C10	−56.9 (2)
C1—C2—C3—C4	1.2 (3)	C6—C5—C11—C14	114.5 (2)
C1—C2—C3—C7	−177.73 (18)	C4—C5—C11—C14	−65.8 (3)
C2—C3—C4—O1	178.34 (17)	C6—C5—C11—C12	−4.3 (3)
C7—C3—C4—O1	−2.8 (3)	C4—C5—C11—C12	175.47 (19)
C2—C3—C4—C5	−4.5 (3)	C6—C5—C11—C13	−122.4 (2)
C7—C3—C4—C5	174.38 (18)	C4—C5—C11—C13	57.3 (3)
O1—C4—C5—C6	−178.28 (17)	C6—C1—C15—S1	−102.63 (19)
C3—C4—C5—C6	4.7 (3)	C2—C1—C15—S1	77.0 (2)
O1—C4—C5—C11	1.9 (3)	C16—S1—C15—C1	61.45 (16)
C3—C4—C5—C11	−175.14 (19)	C15—S1—C16—C17	81.49 (15)
C2—C1—C6—C5	−1.6 (3)	N2—N1—C17—O2	−174.54 (18)
C15—C1—C6—C5	178.00 (18)	N2—N1—C17—C16	7.7 (3)
C4—C5—C6—C1	−1.5 (3)	S1—C16—C17—O2	−87.7 (2)
C11—C5—C6—C1	178.29 (18)	S1—C16—C17—N1	89.99 (19)
C2—C3—C7—C8	3.3 (3)	N1—N2—C18—C20	2.5 (3)
C4—C3—C7—C8	−175.55 (18)	N1—N2—C18—C19	−177.17 (17)
C2—C3—C7—C9	−115.9 (2)		

### Hydrogen-bond geometry (Å, °)

**Table:**

*D*—H···*A*	*D*—H	H···*A*	*D*···*A*	*D*—H···*A*
N1—H1···O2^i^	0.88	2.10	2.940 (2)	159

Symmetry codes: (i) −*x*+1, −*y*+1, −*z*+1.
